# Durability of Polymer-Modified Asphalt Mixture with Wasted Tire Powder and Epoxy Resin under Tropical Climate Curing Conditions

**DOI:** 10.3390/polym15112504

**Published:** 2023-05-29

**Authors:** Kyung-Nam Kim, Tri Ho Minh Le

**Affiliations:** 1 Department of Highway & Transportation Research, Korea Institute of Civil Engineering and Building Technology, 283 Goyangdae-Ro, Ilsanseo-Gu, Goyang-si 10223, Gyeonggi-Do, Republic of Korea; kimkyungnam@kict.re.kr; 2 Faculty of Civil Engineering, Nguyen Tat Thanh University, 300A Nguyen Tat Thanh Street, District 4, Ho Chi Minh City 70000, Vietnam

**Keywords:** polymer-modified asphalt binder, epoxy resin, crumb rubber powder, SBS, asphalt concrete, tropical climate curing, overload, durability, sustainability

## Abstract

The quality of pavements in tropical climates is negatively affected by the frequent wet and dry cycles during the rainy season, as well as by issues related to overloading from heavy trucks and traffic congestion. Contributing to this deterioration are factors such as acid rainwater, heavy traffic oils, and municipal debris. In light of these challenges, this study aims to assess the viability of a polymer-modified asphalt concrete mixture. This study investigates the feasibility of a polymer-modified asphalt concrete mixture with the addition of 6% crumb rubber powder from waste car tires and 3% epoxy resin to counter the harsh conditions of tropical climate weather. The study involved subjecting test specimens to five to 10 cycles of contaminated water (100% rainwater + 10% used oil from trucks), curing for 12 h, and air drying in a chamber of 50 °C for 12 h to simulate critical curing conditions. The specimens underwent fundamental laboratory performance tests such as the indirect tensile strength test, dynamic modulus test, four points bending test, and Cantabro test, as well as the double load condition in the Hamburg wheel tracking test to determine the effectiveness of the proposed polymer-modified material in actual conditions. The test results confirmed that the simulated curing cycles had a critical impact on the durability of the specimens, with the greater curing cycles leading to a significant drop in the strength of the material. For example, the TSR ratio of the control mixture dropped from 90% to 83% and 76% after five and 10 curing cycles, respectively. Meanwhile, the modified mixture showed a decrease from 93% to 88% and 85% under the same conditions. The test results revealed that the effectiveness of the modified mixture outperformed the conventional condition in all tests, with a more prominent impact observed under overload conditions. Under double conditions in the Hamburg wheel tracking test and a curing condition of 10 cycles, the maximum deformation of the control mixture sharply increased from 6.91 to 22.7 mm, whereas the modified mixture increased from 5.21 to 12.4 mm. Overall, the test results confirm the durability of the polymer-modified asphalt concrete mixture under harsh tropical climate conditions, promoting its application for sustainable pavements, especially in Southeast Asian countries.

## 1. Introduction

Asphalt concrete is a widely used material in road construction due to its excellent properties such as durability, flexibility, and resistance to weathering [1,2]. However, the pavement quality in tropical climate conditions is adversely affected by the frequent cycles of wet and dry conditions during the rainy season, coupled with overloading issues from heavy trucks and traffic congestion [3]. Contaminated water from acid rains, heavy traffic oils, and municipal debris also contribute to the deterioration of pavement quality [4,5]. Therefore, developing an asphalt concrete mixture with improved durability and sustainability is of paramount importance [6,7].

Tropical climate conditions can have a significant impact on the performance of asphalt concrete pavements due to the frequent cycles of wet and dry conditions during the rainy season, as well as the high temperatures and humidity [8,9]. Recent studies have focused on developing asphalt concrete mixtures that can withstand these harsh conditions and improve the durability and sustainability of pavements in tropical regions [6,7].

One approach that has been explored is the use of modified asphalt binders [10]. For example, some studies have investigated the use of Sasobit and Sasobit-RE (recovered from waste plastics) additives to improve the resistance of asphalt binders to high-temperature cracking and aging in tropical climates [11]. Other studies have investigated the utilization of waste cooking oil as a potential modifier for asphalt binders [12]. In addition to modified asphalt binders, the use of aggregate gradation and size has also been studied. Research has shown that using finer aggregate gradations and smaller aggregate sizes can improve the stability and resistance to the rutting of asphalt concrete mixtures in tropical climates [13]. Furthermore, the use of recycled aggregates and waste materials, such as crushed glass and ceramic, has also been investigated as a sustainable alternative to traditional aggregates [14,15,16]. To improve the durability of asphalt concrete pavements in tropical climates, some studies have focused on the use of asphalt mixtures with high-strength properties [17,18]. For example, research has shown that using high-performance fiber-reinforced asphalt concrete (HPFRAC) can improve the resistance of pavements to cracking and rutting [19,20]. Other studies have explored the use of hybrid fibers, such as carbon and basalt fibers, to enhance the mechanical properties of asphalt concrete mixtures in tropical regions [21]. Overall, recent findings have demonstrated the importance of developing asphalt concrete mixtures that can withstand the harsh conditions of tropical climates [22]. By exploring the use of modified asphalt binders, aggregate gradation and size, and high-strength materials, researchers have made significant progress in improving the durability and sustainability of pavements in tropical regions [22,23].

However, despite the significant progress made in the development of asphalt concrete mixtures for tropical climate conditions, several limitations remain. One key limitation is the lack of research on the use of alternative materials in asphalt mixtures, particularly in the use of various additives such as polymers, crumb rubber, and fibers. Additionally, most studies have focused on the short-term performance of these mixtures, with little attention paid to their long-term durability under extreme tropical climate conditions [22]. There is a need for further investigation into the use of other alternative materials that can provide similar or even better performance.

One promising approach is the use of polymer-modified asphalt binders [10,24,25], which can enhance the mechanical properties of the mixture, such as stiffness, strength, and resistance to deformation [26,27]. The addition of crumb rubber powder from waste car tires and epoxy resin has also been investigated to further improve the properties of the mixture [25,28,29]. Recent research has confirmed the feasibility of crumb rubber application into the asphalt concrete for sustainability development purposes [30,31]. Previous studies have reported on the effectiveness of polymer-modified asphalt concrete mixtures in practice. For instance, the use of styrene–butadiene–styrene (SBS) polymer-modified asphalt binder was found to improve the resistance to rutting and cracking under high temperatures in arid and semi-arid regions [32,33]. However, the durability and performance of these mixtures under tropical climate conditions have not been extensively studied.

**Figure 1:**
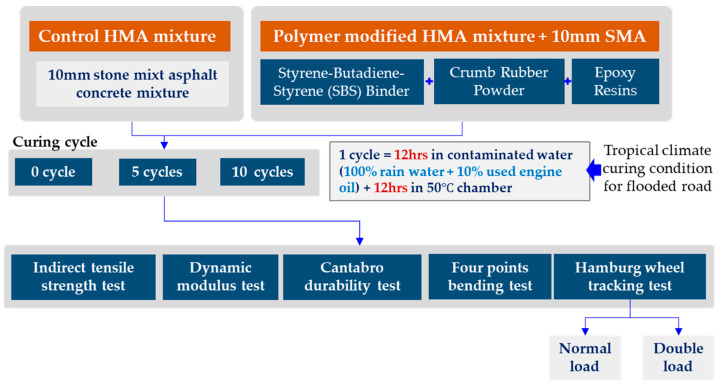
Research flowcharts.

The pavement quality in tropical climates can be compromised by various factors, including acid rain, traffic oils, and municipal debris, which can lead to pavement deterioration [34]. Therefore, this research aims to investigate the feasibility of using a polymer-modified asphalt concrete mixture to improve the durability of pavement under tropical climate conditions. The proposed mixture includes 6% crumb rubber powder from waste car tires and 3% epoxy resin. Test specimens will be exposed to five to 10 cycles of contaminated water (100% rainwater + 10% used oil from trucks), curing for 12 h and air drying at 50 °C for 12 h to simulate critical curing conditions. The specimens will then undergo fundamental laboratory performance tests, such as the indirect tensile strength test, dynamic modulus test, and Cantabro test, to evaluate their mechanical properties. In addition, the double load condition in the Hamburg wheel tracking test will be conducted to determine the effectiveness of the polymer-modified material in actual conditions. Overall, this study aims to provide insights into the feasibility of using the proposed polymer-modified asphalt concrete mixture for sustainable pavements in tropical climates. The general flow of the research is presented in Figure 1.

## 2. Materials and Methods

### 2.1. Materials

#### 2.1.1. Aggregate

The aggregate used in this study is the 10 mm nominal maximum aggregate size (NMAS) for stone matrix asphalt (SMA). The aggregate was sourced from a Korean quarry plant and was in compliance with Korean Industrial Standards. The physical properties of the aggregate were determined in accordance with the Korean Standards for aggregates. The aggregate was cleaned and dried in an oven at a temperature of 110 °C for 24 h before use in the preparation of the asphalt concrete mixtures. The sieve analysis was conducted in accordance with the Korean Standards for aggregates to ensure the uniformity of the aggregate used in the study as shown in Table 1. The quality of the aggregate is presented in Table 2 below.

#### 2.1.2. Polymer-Modified SBS Asphalt Binder

Polymer-modified asphalt binders are known for their improved rheological properties, such as increased stiffness, reduced deformation, and enhanced durability. In this study, the asphalt binder used was a polymer-modified styrene–butadiene–styrene (SBS) binder (Table 3), which is a commonly used polymer in asphalt modification due to its superior properties. The SBS polymer modification was added to the asphalt binder in the form of pellets during the mixing process. The amount of SBS polymer added to the asphalt binder was based on the recommendations of Korean road and pavement experts and the author team’s experience in trial work. With regard to the additive used in this research, the properties of crumb rubber powder and epoxy resin are summarized in Table 4 and Table 5, respectively. First, 6% crumb rubber powder from waste car tires was added to the asphalt mixture to improve its performance. The crumb rubber powder was treated with a hot water bath and dried before it was introduced into the mixture. The hot water bath was used to remove any residual contaminants and soften the rubber, making it more workable. The crumb rubber powder was then mixed with the asphalt binder and aggregates in the proper ratio. Moreover, 3% epoxy resin was added to the asphalt mixture to enhance its strength and durability. Epoxy resin is a thermosetting polymer that can improve the mechanical properties of asphalt concrete. It forms a strong bond with the asphalt binder and aggregates, which enhances the mixture’s strength and durability. The epoxy resin was added to the mixture during the mixing process and mixed thoroughly with the other materials. The general properties of the modified asphalt binder with SBS and additives are presented in Table 6.

#### 2.1.3. Mix Design

This research utilized the Superpave mix design approach to determine the optimal asphalt binder content and aggregate gradation, taking into account traffic volume, climatic conditions, and desired pavement performance [47,48,49]. The mix design aimed for a target air void content of 4%, with a minimum VMA of 18% and VFA of 75%. The final mix design consisted of a 6.5% SBS-modified asphalt binder, which includes 6% crumb rubber powder and 3% epoxy resin, 18.5% fine aggregate, and 75% coarse aggregate by weight. In this research, it is important to note that the SBS content in the modified asphalt binder was 2.5% by weight of the asphalt binder. This proportion was carefully selected to achieve the desired performance characteristics of the modified asphalt mixture.

In the laboratory, the mixing process for the polymer-modified asphalt concrete mixture involved several steps. Initially, the aggregate was heated to a temperature of 160 °C using a dryer drum. Subsequently, the SBS polymer, crumb rubber powder, and epoxy resin were introduced to the heated aggregate and mixed for approximately 1–2 min. The temperature of the mixture was closely monitored and maintained within the range of 150–160 °C. Following the mixing process, the mixture was compacted using the Superpave gyration compactor method [50].

Considering the curing condition of the epoxy resins, the epoxy resin used in this study is thermosetting and requires heat to initiate the curing process. During the mixing of the asphalt mixture, the epoxy resin is added as a liquid component, which disperses and coats the aggregate particles. Once the HMA mixture is compacted and subjected to the asphalt production temperature, the heat triggers the curing process of the epoxy resin. This results in the formation of a durable cross-linked network within the modified asphalt binder, enhancing its cohesive properties and resistance to crack propagation.

#### 2.1.4. Curing Conditions

The test specimens were prepared by subjecting them to a curing process that simulated critical field conditions. This was achieved by subjecting the specimens to 5 to 10 cycles of curing with contaminated water and air drying. The contaminated water used for curing was a mixture of 100% rainwater and 10% used oil from trucks. In the mixing process of the used engine oil in the contaminated water, constant agitation was employed to achieve a uniform dispersion of the oil and water components. The use of constant agitation ensured thorough mixing, facilitating the formation of a homogeneous mixture. To further enhance the miscibility of the oil and water, surfactants were utilized during the emulsification process. These surfactants played a crucial role in reducing interfacial tension and promoting the formation of stable oil-in-water emulsions. The surfactant content used in the emulsification process was 1% by weight of the total volume of the contaminated water and oil mixture. This concentration was determined through preliminary experiments and optimization studies to achieve a balance between effective emulsification and minimizing potential interference with the system. By employing this approach, the specimens were uniformly treated, and the oil was uniformly dispersed in the contaminated water throughout the entire experimental process. This method of mixing and the inclusion of surfactants ensured consistent and reliable results in the evaluation of the contaminated water samples.

**Figure 2:**
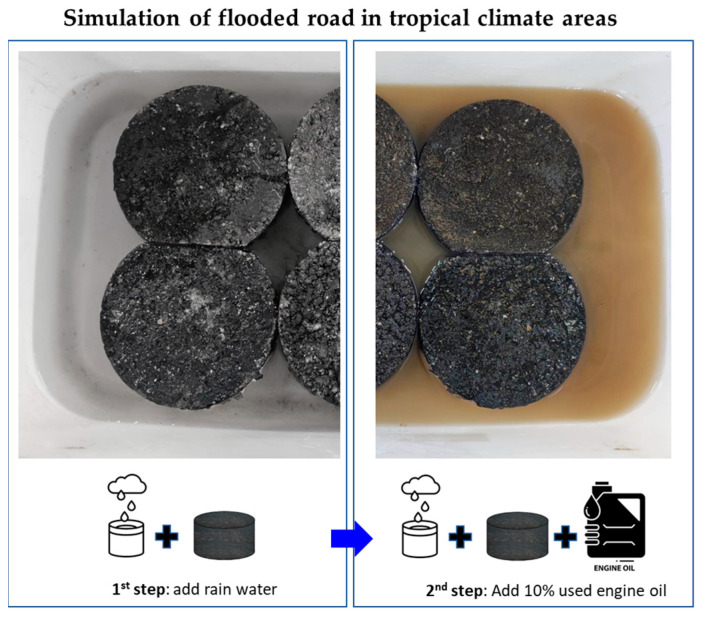
Fabricating of curing condition for the research.

With regard to the cyclic curing process, each cycle involved a 12 h period of curing in the contaminated water followed by 12 h of air drying in a chamber maintained at a temperature of 50 °C. This curing cycle was developed to simulate the critical curing conditions experienced by asphalt pavements in tropical climates during the rainy season. During this season, the pavement is dry from early morning to noon, with pavement temperatures reaching up to 50 °C. Then, rain typically starts in the afternoon (around 3 pm) and lasts until nighttime. The proposed curing cycle subjects the test specimens to cycles of contaminated water curing and air drying at high temperatures to mimic the wetting and drying cycles that occur during the rainy season. The 5 and 10 cycles of treatments in this laboratory experiment are designed to simulate accelerated aging and evaluate the short-term performance and durability of the modified asphalt mixtures. These accelerated aging cycles are not intended to precisely mimic the exact number of years of field weathering but rather to provide initial indications of the performance trends and relative durability of the modified asphalt mixtures. As shown in Figure 2, the addition of used oil to the rainwater helps to simulate the presence of surface contamination on the pavement surface. By subjecting the test specimens to these conditions, the study aims to evaluate the performance of the proposed polymer-modified asphalt concrete mixture under realistic environmental conditions.

### 2.2. Methods

#### 2.2.1. Tensile Strength Ratio Test

**Figure 3:**
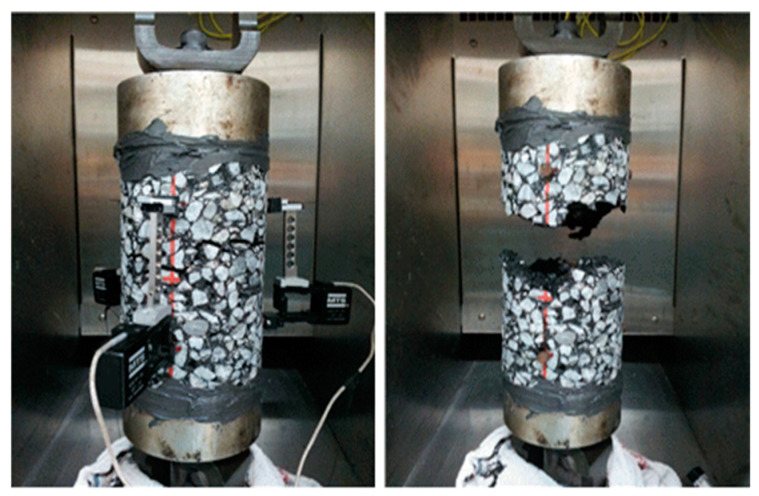
Indirect tensile strength test.

The indirect tensile strength (ITS) test is a common method used to evaluate the mechanical properties of asphalt concrete. In this study, the ITS test was conducted according to the ASTM D6931 standard to determine the maximum tensile strength of the asphalt concrete mixture [51]. The test was performed using a universal testing machine (UTM) at a controlled rate of deformation (see Figure 3). The standard specimen size for TSR testing is 100 mm in diameter × 60 mm in height. The specimens were loaded diametrically at a constant rate of deformation until failure. The tensile strength ratio (TSR) was also calculated to determine the resistance of the mixture to permanent deformation. It should be noted that both dry and wet conditions were used to calculate the TSR ratio. The aging process was carried out by subjecting the specimens to a combination of high temperature and air for 3 h, followed by a water immersion process at 80 °C for 24 h. The TSR ratio was calculated for both dry and wet conditions at a temperature of 25 °C and 60 °C. The loading rate for the TSR test of asphalt concrete is specified as 50 mm/min. This loading rate ensures a consistent and controlled application of load during the test, allowing for accurate measurement of the tensile strength properties of the asphalt concrete specimen. The TSR ratio values were then compared with the standard requirements to assess the performance of the asphalt concrete mixture. The results from the ITS test and TSR were used to evaluate the effectiveness of the proposed polymer-modified asphalt concrete mixture in improving the mechanical properties and resistance to deformation.

#### 2.2.2. Dynamic Modulus Test

**Figure 4:**
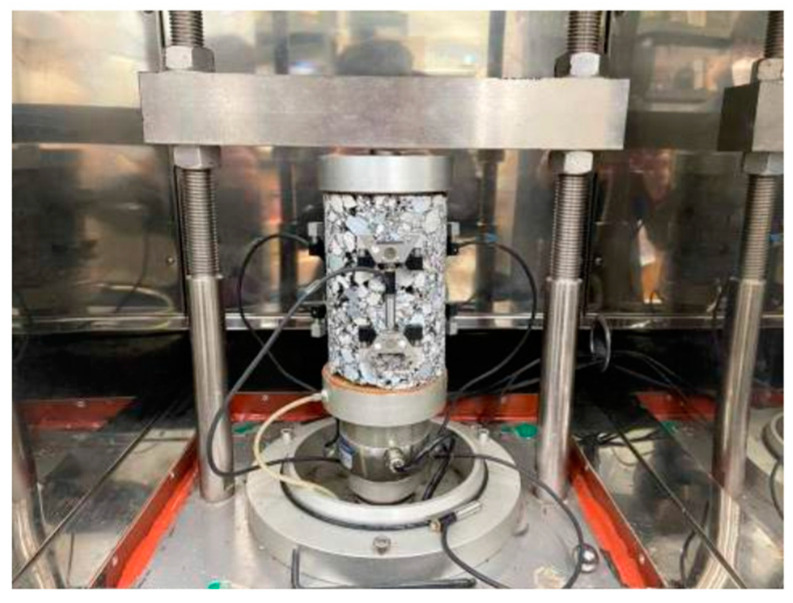
Dynamic modulus test apparatus.

The dynamic modulus test is a commonly used method to evaluate the stiffness and viscoelastic behavior of asphalt concrete under varying loading conditions. In this study, the dynamic modulus test was conducted according to the AASHTO TP 62 [52] standard using a universal testing machine (UTM) equipped with a dynamic modulus apparatus (see Figure 4). The standard specimen size for dynamic modulus testing is cylindrical with a diameter of 100 mm and a height of 150 mm. To examine the characteristics of the mixtures across a range of temperatures, the test was conducted at various temperatures, spanning from −10 °C to 54 °C. Additionally, the loading frequency was segmented from 25 Hz to 0.1 Hz. This approach allowed for a comprehensive analysis of the mixtures’ behavior under different temperatures and loading conditions. The specimens were tested at a range of temperatures and frequencies to determine the complex modulus (E*) and phase angle (δ). The results were used to develop a master curve of E* as a function of temperature using the time–temperature superposition (TTS) principle. The master curve provided information about the stiffness of the asphalt concrete over a wide range of temperatures and loading frequencies. The results from the dynamic modulus test were used to evaluate the effectiveness of the proposed polymer-modified asphalt concrete mixture in improving the stiffness and viscoelastic behavior of the asphalt concrete.

The relationship between viscosity and temperature susceptibility is described by Equation (1) [52], where the logarithm of viscosity (η) is expressed as a function of temperature ($T_{R}$). The equation includes parameters A and VTS, representing the intercept and slope of this relationship, respectively. To further analyze the impact of viscosity–temperature susceptibility, Equation (2) is developed [52]. This equation introduces a transition function that utilizes VTS to divide the viscosity at a specific temperature by the viscosity at each temperature. It includes constants and reference temperatures, denoted as c, $T_{R}$, and ${\left(T_{R}\right)}_{0}$, respectively. These equations provide a mathematical framework for understanding the relationship between viscosity and temperature in the context of the given study or analysis. They allow for the exploration of viscosity variations with changing temperatures and the calculation of transitions between different temperature points based on their respective viscosities.

(1) $$\log\left(\log\eta \right)=A+\mathrm{VTS}\left[\log\left(T_{R}\right)\right]$$

(2) $$\log\left(T\right)=c\left(10^{A+\mathrm{VTS}\left[\log\left(T_{r}\right)\right]}-10^{A+\mathrm{VTS}\left[\log{\left(T_{R}\right)}_{0}\right]}\right)$$

#### 2.2.3. Cantabro Test

**Figure 5:**
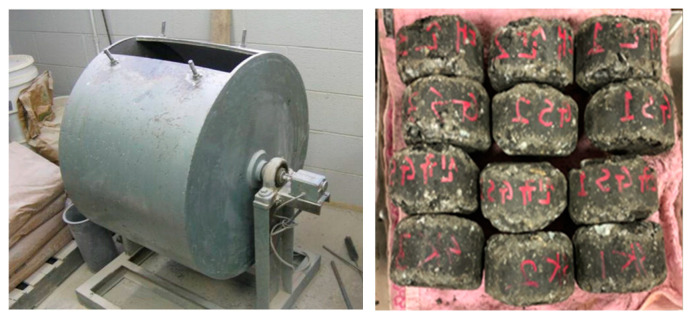
Cantabro test of specimens.

The Cantabro test is used to evaluate the resistance of asphalt concrete to breakage due to repeated traffic loads. In this study, the test was performed according to the ASTM D6927 standard [53]. The Cantabro test employs cylindrical specimens with a diameter of 100 mm and a height of 50 mm. The test specimen was placed in a special device, and steel balls were dropped repeatedly onto the surface of the specimen for a specified number of times as presented in Figure 5. Subsequently, the specimen was placed into the testing drum, excluding the presence of a steel ball. The machine was then operated at speeds ranging from 30 to 33 rpm, completing a total of 300 rotations at a room temperature of 28 ± 2 °C. Following the completion of this process, the sample was carefully extracted from the machine, and its weight was measured to determine the level of abrasion. The loss of mass of the specimen was recorded after each set of drops. The result from the Cantabro test was used to evaluate the durability of the asphalt concrete mixture and its ability to withstand repeated traffic loads.

#### 2.2.4. Four-Point Bending (4 PB) Test

**Figure 6:**
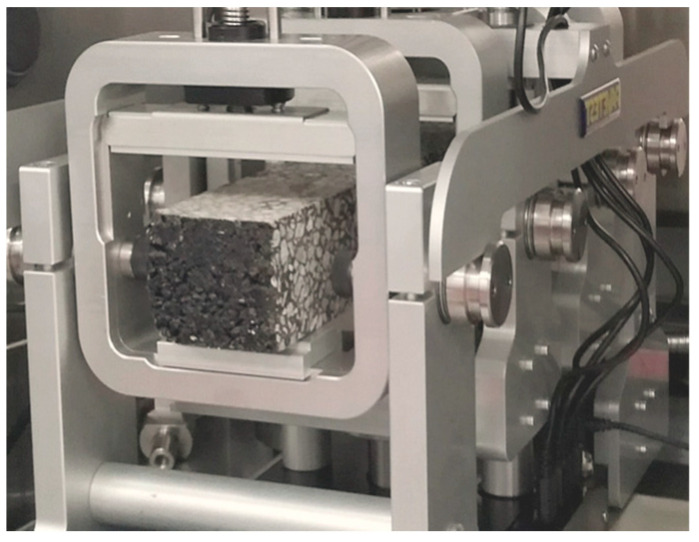
Testing apparatus of 4 PB test.

The four-point bending test is a widely employed method for assessing the flexural properties of asphalt materials in accordance with ASTM D6272 [54]. It plays a significant role in characterizing the behavior of asphalt under bending loads, which is vital for understanding its structural performance in real-world applications. The test involves subjecting a rectangular asphalt specimen to a bending moment by applying forces at two points along the specimen’s length while supporting it at two additional points as presented in Figure 6. This configuration creates a bending moment distribution within the specimen, simulating the conditions it would experience in a pavement structure. The specimens utilized in the four-point bending beam test were of dimensions 305 mm long × 45 mm wide × 50 mm depth. Typical test parameters include a loading rate of 5 mm/min and a span length of 150 mm. By measuring the load and the resulting deflection, the test provides valuable information about the asphalt’s stiffness, strength, and ability to resist cracking under bending stresses.

#### 2.2.5. Hamburg Wheel Tracking (HWT) Test

**Figure 7:**
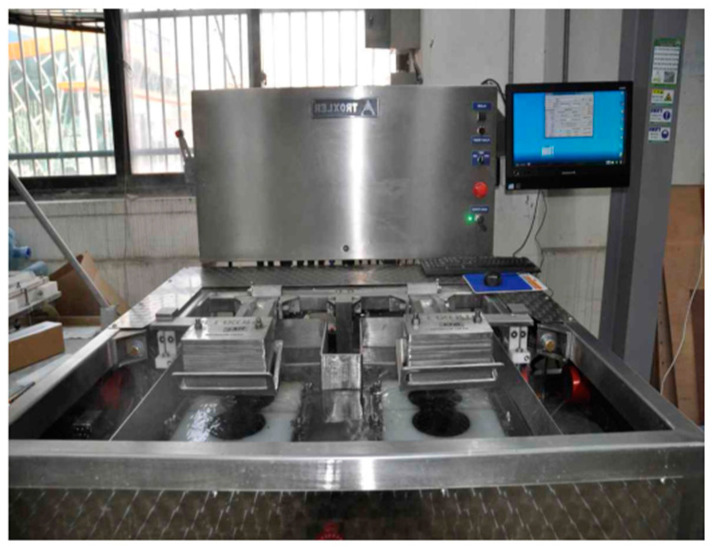
HWT results.

The HWT test is used to evaluate the rutting resistance of asphalt concrete mixtures under traffic loading and environmental conditions. In this study, the HWT test was performed according to AASHTO T324 [55] standard using a loaded wheel (see Figure 7) with a 700 mm diameter. The HWT test utilizes specimens with dimensions of approximately 150 mm in diameter and 62 mm in height. The test specimens were conditioned at 60 °C for 24 h and then loaded for a specified number of cycles. A steel wheel, measuring 45 mm in width, applies a load of 700 ± 5 N to the specimen. The wheel undergoes 50 passes per mins across each specimen, with the highest velocity of 340 ± 5 mm/sec at the center of the specimens. The rut depth was measured every 1000 cycles to determine the resistance to permanent deformation. The settlement amount of the HMA mixture is mandated by the US State Department of Transportation to ensure its performance, and it should not exceed 20 mm after 20,000 cycles according to the reference. The test was conducted under the double load condition to simulate the actual traffic conditions and to determine the effectiveness of the proposed polymer-modified material in actual conditions [56]. The results from the HWT test were used to evaluate the rutting resistance of the mixture and to compare the performance of the polymer-modified mixture with that of the conventional asphalt concrete mixture.

## 3. Results and Discussions

### 3.1. Tensile Strength Ratio Test

**Figure 8:**
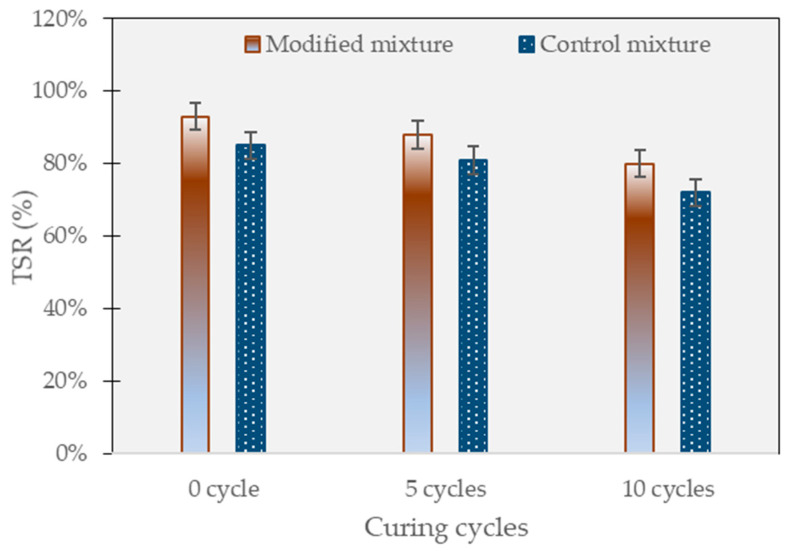
TSR test results.

In this study, the tensile strength ratio (TSR) was used to evaluate the resistance of the control and modified SMA mixtures to permanent deformation under critical curing conditions. The specimens were subjected to five to 10 cycles of contaminated water curing and air drying in a chamber of 50 °C, simulating the critical curing conditions that occur during the rainy season in tropical climate countries. As shown in Figure 8, the results showed that the modified mixture had a higher TSR than the control mixture, indicating greater resistance to permanent deformation. This can be attributed to the addition of the polymer modifier, crumb rubber, and epoxy resin, which improved the binder properties and increased the strength and elasticity of the mixture.

The modified mixture exhibited a TSR of 88% after five cycles and 80% after 10 cycles, while the control mixture showed a TSR of 81% after five cycles and 72% after 10 cycles. This indicates that the polymer-modified mixture has better resistance to permanent deformation under simulated critical curing conditions. The coating and water-proof properties of the polymer-modified mixture may have contributed to its ability to withstand the impact of contaminated curing conditions. Additionally, the acid resistivity of the polymer-modified mixture may have played a role in its improved TSR performance. The incorporation of crumb rubber and epoxy in the polymer-modified mixture also likely contributed to its enhanced resistance to deformation. Furthermore, the use of contaminated water in the curing process simulates the harsh conditions of the real-world environment and provides a more accurate representation of the performance of the mixtures. Overall, the results suggest that the polymer-modified mixture is better suited to withstand the harsh curing conditions commonly experienced during the rainy season in tropical climate countries.

### 3.2. Dynamic Modulus Test

**Figure 9:**
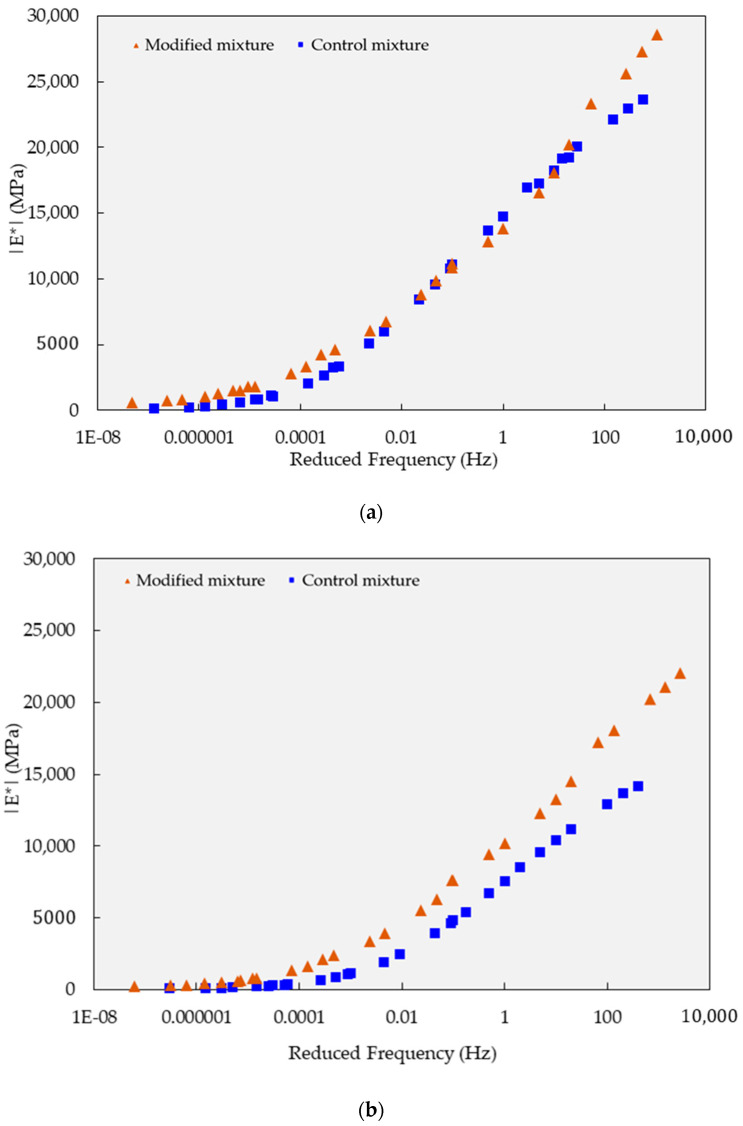
Dynamic modulus test results on mixtures based on the semi-log scale and focusing on the high-frequency range: (**a**) five-cycle curing condition; and (**b**) 10-cycle curing condition.

**Figure 10:**
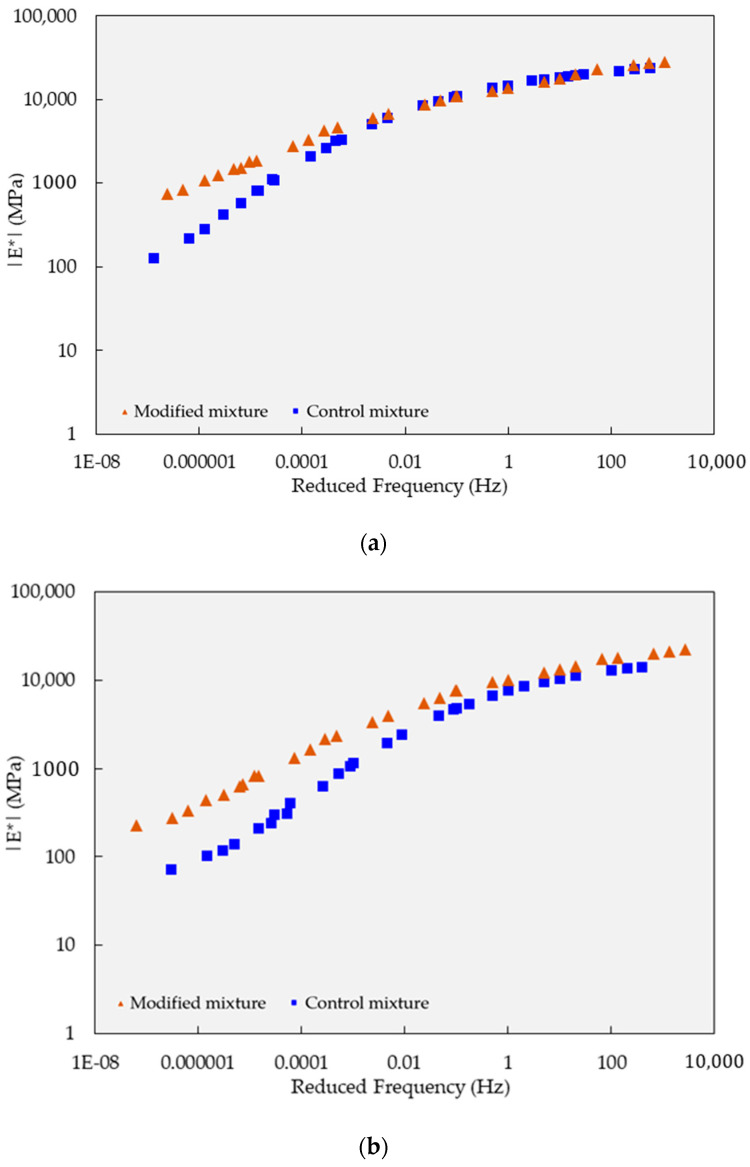
Dynamic modulus test results on mixtures based on the semi-log scale and focusing on the high-frequency range: (**a**) five-cycle curing condition; and (**b**) 10-cycle curing condition.

In the dynamic modulus test, the experimental data were analyzed to generate the master curve, as presented in Figure 9 and Figure 10. In general, both types of mixtures displayed similar behavior characteristics in terms of modulus of elasticity concerning temperature and load cycle, except under long-term curing conditions.

Further investigating the behavior of the test mixture after the curing process, the polymer-modified mixture demonstrated a significantly higher modulus of elasticity compared to the reference mixture in the high-temperature range, particularly in the high and slow load frequency region.

The reinforcement effectiveness is more prominent when specimens were subjected to 10 curing cycles. After 10 curing cycles, the dynamic modulus values at the high-frequency range indicate that the modified specimens exhibited a significantly higher value of 22,084 MPa compared to the control specimens, which had a dynamic modulus of 14,178 MPa (see Figure 9b).

Considering the behavior of samples under a low-frequency zone, the dynamic modulus test results after 10 curing cycles at the low-frequency range revealed a substantial difference in the dynamic modulus between the modified and control specimens. As presented in Figure 10b, the modified specimens exhibited a significantly higher dynamic modulus of approximately 226 MPa, representing an increase of about 213% compared to the control specimens, which had a dynamic modulus of only 72 MPa. This implies that a polymer-modified mixture has superior resistance to plastic deformation, which is an essential factor in the durability and resistance to permanent deformation of asphalt pavements.

Additionally, the polymer-modified mixture also exhibited a remarkably higher modulus of elasticity than the reference mix under high load cycles, indicating a potential reduction in susceptibility to low-temperature-induced cracking. This finding suggests that a polymer-modified mixture may offer improved resistance against temperature-induced cracking, which is crucial for asphalt pavements subjected to heavy traffic and fluctuating environmental conditions. Overall, the higher modulus of elasticity observed in a polymer-modified mixture indicates its enhanced ability to resist plastic deformation under high-temperature conditions, making it particularly suitable for applications where durability and resistance to permanent deformation are crucial factors.

### 3.3. Cantabro Test

The Cantabro test findings are summarized in Table 7. To calculate the loss rate brought on by the Cantabro procedure, the dry weight of each Marshall sample was determined prior to testing, and the final mass after testing was noted. Following the test findings, all test samples had severe surface damage, with observable damaged ratios (%) across all circumstances. While the reference combination exhibited a loss rate of up to 12.87 and 21.35% during the short (five) and long (10) curing cycles, respectively, the modified combination had a loss rate of 8.15–15.97% under the same testing settings. The tightening effect generated by the addition of polymer and additives increased the modified mixture’s efficacy. This indicates that incorporating polymer and additives into the modified mixture has a positive effect on its durability, as evidenced by the reduced loss rate compared to the reference mixture. The stiffening effect of the modified mixture due to the inclusion of polymers and additives also contributes to its improved effectiveness, which is consistent with the findings of the previous tests. The higher durability of the modified mixture can be attributed to its resistance to surface damage caused by the Cantabro procedure, which demonstrates its ability to withstand the critical curing conditions used in the study. These results suggest that the modified mixture may be more suitable for use in tropical regions with high heat and rainy conditions, where the pavement may be subjected to extreme curing conditions.

### 3.4. Four-Point Bending Test

**Figure 11:**
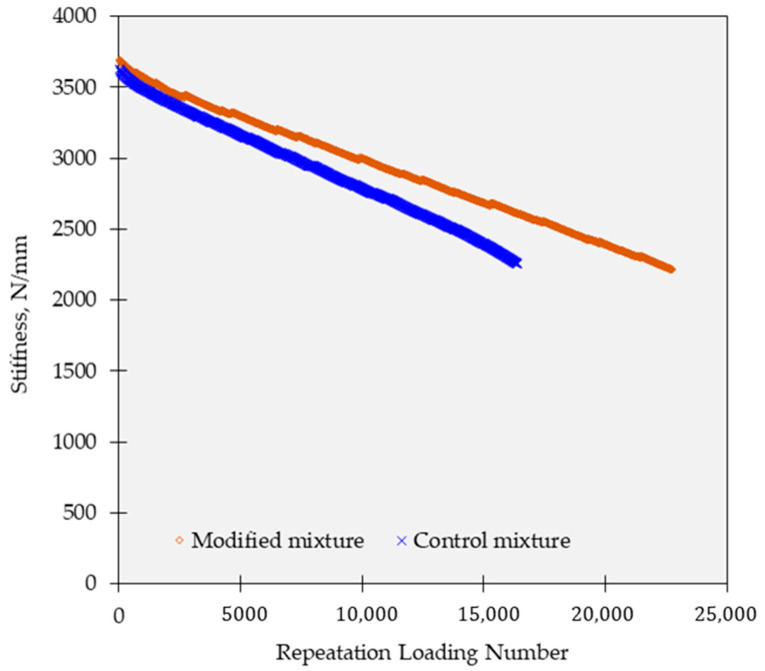
Four-point bending test results of samples subjected to 10 cycles.

In the four-point bending test, both the control and modified specimens exhibited a gradual reduction in stiffness as the load was applied. This behavior is indicative of the deformation and response of the pavement materials under loading conditions. However, a notable difference was observed between the control and modified specimens. As depicted in Figure 11, the control specimen experienced a sudden failure at 16,500 test cycles, indicating its limited ability to withstand repeated loading. On the other hand, the modified mixture showed improved performance, surpassing 22,000 cycles before reaching failure. This finding suggests that the incorporation of the polymer modification in the mixture enhanced its fatigue resistance and prolonged its service life under repeated loading conditions. The improved performance of the modified mixture, withstanding higher test cycles before failure compared to the control specimen, highlights the effectiveness of the polymer modification in enhancing the durability and fatigue resistance of the asphalt concrete.

### 3.5. Hamburg Wheel Tracking (HWT) Test

**Figure 12:**
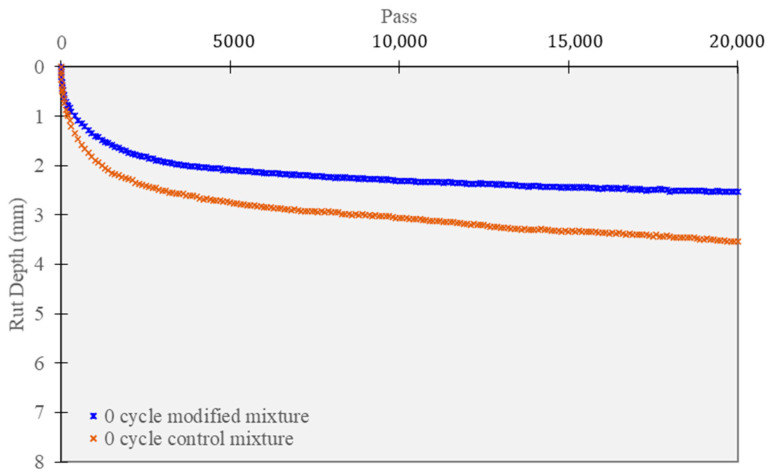
Hamburg wheel tracking test of unconditioned specimens.

The HWT test outcomes are illustrated in Figure 12 for the mixtures tested under 0-cycle curing conditions. Initially, under normal curing conditions (0 cycles), all mixtures displayed a sharp settlement during the first 2000 cycles, followed by a gradual increase in settlement rate. The test findings indicate that the polymer-modified mixture has superior rutting resistance. Specifically, the settlement of the modified mixture was less than 2.52 mm at 20,000 cycles, while the reference mixture’s settlement was higher than 3.53 mm. In addition, the stripping point phenomenon was not observed in any of the mixtures.

**Figure 13:**
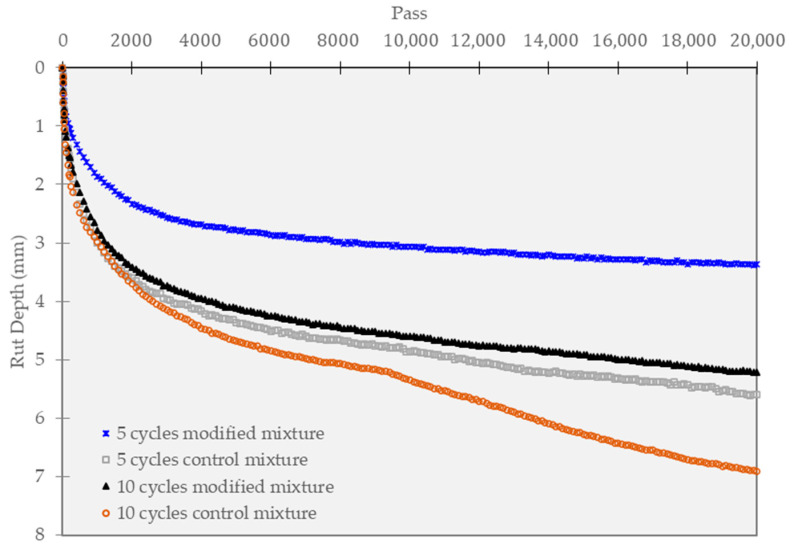
Hamburg wheel tracking test of conditioned specimens.

Regarding the effect of curing conditions under standard HWT loads, the test results indicate that the reference mixture suffered a significant increase in rutting values compared to the polymer-modified mixture. Analysis of the rutting depth (mm) value, when the wheel load reached 20,000 cycles, showed that the addition of polymer and additives effectively reduced plastic deformation. For instance, as presented in Figure 13, during short-term curing, the reference mixture experienced approximately 5.6 mm of plastic deformation, while the modified mixture experienced less than 3.4 mm.

Under long-term curing conditions of 10 cycles, the maximum deformation of the control mixture sharply increased from 5.6 to 6.91 mm, while the modified mixture increased from 3.4 to 5.21 mm. Moreover, as it is a water immersion test, the addition of polymer accompanied by the appropriate content of CRP and epoxy was determined to improve moisture resistance. As a result, the asphalt pavement materials with CRP (6%) + Epoxy (3%) showed the highest resistance to stripping.

**Figure 14:**
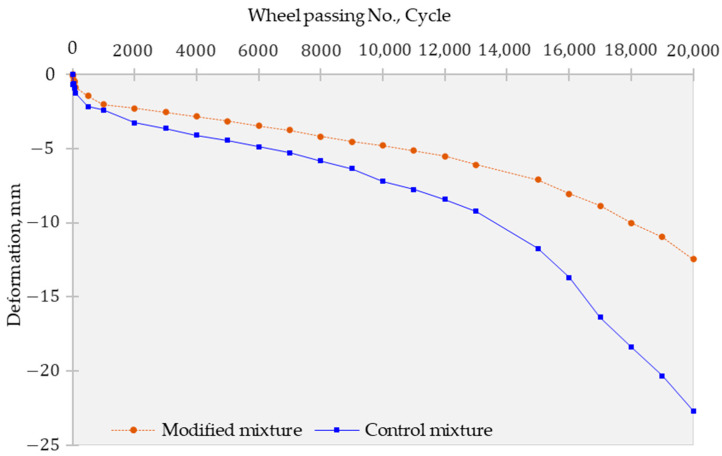
Double-load HWT test of 10-cycle conditioned specimens.

Additionally, to simulate the overloading effect in tropical climate conditions, the Hamburg wheel tracking test was conducted at an increased load of two times the standard value of 7054.5 N, as shown in Figure 14. The test results revealed that all settlement amounts were less than 20 mm in the modified mixture (12.4 mm), meeting the criteria when the load was significantly increased. However, it should be noted that the settlement growth rate increased by an average of 2.5 times compared to the conventional loading condition, indicating the potential risk of plastic deformation failure under this condition. In contrast, the reference mixture was significantly impacted by both contaminated curing conditions and the overload effect, with a sudden stripping point observed at 12,000 and a maximum rutting value of more than 22.7 mm.

The Hamburg wheel tracking test results indicate the significant role of the polymer-modified mixture in enhancing the rutting resistance and durability of the asphalt pavement materials under different curing conditions and loading scenarios. The incorporation of polymer and additives into the mixture has contributed to the tightening effect, which led to a significant reduction in plastic deformation and settlement amounts. In addition, the water resistance and acid resistance of the mixture were improved, as a result of the coating and water-proofing effect of the polymer and the proper content of crumb rubber powder and epoxy. The findings suggest that the use of polymer-modified asphalt containing crumb rubber powder and epoxy can be an effective strategy for improving the long-term performance and sustainability of asphalt pavements, especially under harsh environmental and traffic conditions.

Based on the findings from this research, the results suggest the great potential of modified asphalt binder for the durability improvement of asphalt concrete in tropical climate conditions. This can be mainly attributed to the component constituted in the asphalt binder. The modified hot mix asphalt (HMA) mixture comprises three key components: styrene–butadiene–styrene (SBS), crumb rubber powder (CRP), and epoxy resins. Each of these components plays a crucial role in enhancing the mechanical properties of the mixture, resulting in improved performance characteristics.

SBS, as a thermoplastic elastomer modifier, significantly improves the tensile strength of the asphalt mixture. By enhancing the binder’s elastic behavior and crack resistance, SBS allows the modified HMA to better withstand thermal and mechanical stresses. This increased elasticity reduces the likelihood of cracking and enhances the overall tensile strength of the mixture. The inclusion of crumb rubber powder (CRP) in the HMA mixture offers several benefits, particularly in terms of dynamic modulus. The rubber particles present in CRP act as reinforcing fillers, increasing the stiffness of the mixture. This enhancement in dynamic modulus helps the HMA to better resist permanent deformation under traffic loads, reducing rutting potential and ensuring improved long-term performance of the mixture. Epoxy resins, when incorporated into the modified HMA mixture, provide substantial improvements in damage tolerance. These resins introduce additional cohesion and adhesion properties within the mixture, effectively mitigating crack propagation. Through the formation of a durable cross-linked network within the modified asphalt binder, the epoxy resins significantly enhance the mixture’s resistance to cracking, thereby extending its service life.

The combined effect of SBS, CRP, and epoxy resins in the modified HMA mixture results in a material with enhanced mechanical properties and improved performance characteristics. By improving tensile strength, dynamic modulus, and damage tolerance, these components contribute to a more durable and long-lasting asphalt mixture, capable of withstanding various stressors and extending the lifespan of pavement surfaces.

## 4. Conclusions

This research investigates the use of a polymer-modified asphalt concrete mixture containing 6% crumb rubber powder and 3% epoxy resin to improve pavement durability in tropical climates. The mixture is exposed to five to 10 cycles of contaminated water and undergoes laboratory tests. The findings of this research are summarized as follows:

- The addition of crumb rubber and epoxy likely contributed to the enhanced TSR ratio of the modified mixture. After five cycles, the modified mixture showed a TSR of 91%, and 80% after 10 cycles, compared to the control mixture’s TSR of 81% after five cycles and 72% after 10 cycles. The polymer-modified mixture’s coating and waterproof properties, as well as its acid resistivity, may have contributed to its ability to withstand the impact of contaminated curing conditions.
- The dynamic modulus test results after 10 curing cycles at the low-frequency range indicated a significant difference in the dynamic modulus between the modified and control specimens. The modified specimens showed a considerably higher dynamic modulus of approximately 226 MPa, representing a notable increase of about 213% compared to the control specimens, which had a dynamic modulus of only 72 MPa.
- Both the control and modified mixtures experienced severe surface damage during testing, but the modified mixture had a lower loss rate of 8–15% compared to the control mixture’s loss rate of 12–21%. The addition of polymer and additives to the modified mixture resulted in a tightening effect, improving its durability and resistance to surface damage.
- The modified mixture outperformed the control specimen in the four-point bending test, showing a higher resistance to fatigue failure, withstanding over 22,000 cycles compared to the control’s failure at 16,500 cycles.
- The test results showed that the addition of polymer and additives to the mixture reduced plastic deformation and rutting values. The polymer-modified mixture had better rutting resistance than the reference mixture, with a settlement of less than 2.52 mm at 20,000 cycles compared to the reference mixture’s settlement of more than 3.53 mm. There was no observed stripping point phenomenon in either of the mixtures.
- The modified mixture exhibited a maximum deformation of 5.21 mm, which was less than the control mixture’s maximum deformation of 6.91 mm under long-term curing conditions of 10 cycles. The incorporation of polymer, CRP (6%), and epoxy (3%) improved moisture resistance and increased resistance to stripping.
- The Hamburg wheel tracking test was conducted with an increased load of two times the standard value. Settlement amounts were less than 12.5 mm in the modified mixture, but the settlement growth rate increased by an average of 2.5 times compared to the conventional condition. The reference mixture was significantly impacted by both contaminated curing conditions and the overload effect, with a sudden stripping point observed at 12,000 and a maximum rutting value of more than 22.7 mm.
- The polymer-modified mixture is better suited to withstand the harsh curing conditions typically experienced during the rainy season in tropical climate countries. The research findings are limited to laboratory tests and may not fully reflect the performance of the mixtures in real-world conditions. Therefore, further field trials and long-term monitoring are necessary to validate the results.

## Figures and Tables

**Table 1:** Sieve size gradation.

Sieve Size (mm)	16	13.2	9.5	4.75	2.36	1.18	0.6	0.3	0.15	0.075
Gradation	100	100	96.3	65.8	8.7	4.9	3.1	2.5	1.7	0.6

**Table 2:** Aggregate and mineral filler properties.

Properties	Properties	Value
Aggregate	Relative apparent density [35]	2.71
	Water absorption [35]	0.14%
	Aggregate crushed value [36]	17.5%
	Los Angeles abrasion value [37]	25.8%
	Flakiness and elongation index [38]	12.8%
Mineral Filler	Relative apparent density [39]	2.33
	Moisture content [39]	0.01%

**Table 3:** Properties of SBS polymer.

Property	Value
Tensile Strength	3.5 MPa
Elongation at Break	700%
Softening Point	80–100 °C
Penetration Index	25–45

**Table 4:** Properties of crumb rubber powder.

Property	Value
Mesh Size	30–80 mesh
Specific Gravity	1.12–1.14
Ash Content	<5%
Volatile Content	<2%

**Table 5:** Properties of epoxy resin.

Property	Value
Density	1.1–1.3 g/cm^3^
Tensile Strength	65 MPa
Flexural Strength	95 MPa
Glass Transition Temperature	80–90 °C

**Table 6:** Properties of modified asphalt binder.

Properties	Value
Penetration (1/10 mm) 25 °C [40]	65.8 (1/10 mm)
Softening point (°C) [41]	71.0 °C
Ductility at 5 °C (cm/min) [42]	105 cm/min
Thin film oven (160 °C 300 min) [43]	
Mass loss (%) [43]	0.041%
G*/sinδ; at 76 °C (Original) [44]	1.71 kPa
G*/sinδ at 76 °C (after RTFO) [44]	2.38 kPa
G* × sinδ at 76 °C (after PAV) [45]	1488 kPa
Stiffness at −22 °C [46]	182 MPa
m-value at −22 °C [46]	0.32

**Table 7:** Summary of Cantabro test.

	Specimen Weight (g)	Weight after the Test (g)	Loss Rate (within 20% Based on Drainage)
Controlled mix: 5 cycles	1192.5	1039.0	12.87
Controlled mix: 10 cycles	1192.9	938.21	21.35
Modified mix: 5 cycles	1193.1	1095.8	8.15
Modified mix: 10 cycles	1192.6	1002.1	15.97

## Data Availability

The data that support the findings of this study are available on request.
